# Reproductive factors and subtypes of breast cancer defined by hormone receptor and histology

**DOI:** 10.1038/sj.bjc.6602712

**Published:** 2005-08-02

**Authors:** G Ursin, L Bernstein, S J Lord, R Karim, D Deapen, M F Press, J R Daling, S A Norman, J M Liff, P A Marchbanks, S G Folger, M S Simon, B L Strom, R T Burkman, L K Weiss, R Spirtas

**Affiliations:** 1 Department of Preventive Medicine, Keck School of Medicine, University of Southern California/Norris Comprehensive Cancer Center, Los Angeles, CA, USA; 2 Department of Nutrition Research, University of Oslo, Oslo, Norway; 3 Department of Pathology, Keck School of Medicine, University of Southern California, Los Angeles, CA, USA; 4 Division of Public Health, Fred Hutchinson Cancer Research Center and Department of Epidemiology, School of Public Health and Community Medicine, University of Washington, Seattle, WA, USA; 5 Center for Clinical Epidemiology and Biostatistics and Department of Biostatistics and Epidemiology, University of Pennsylvania, Philadelphia, PA, USA; 6 Department of Epidemiology, Rollins School of Public Health, Emory University, Atlanta, GA, USA; 7 Division of Reproductive Health, Centers for Disease Control and Prevention, Atlanta, GA, USA; 8 Department of Internal Medicine, Karmanos Cancer Institute at Wayne State University, Detroit, MI, USA; 9 Department of Obstetrics and Gynecology, Baystate Medical Center, Springfield, MA, USA; 10 Cancer Centers Branch, National Cancer Institute, Bethesda, MD, USA; 11 Contraception and Reproductive Health Branch, Center for Population Research, National Institute of Child Health and Human Development, NIH, DHHS, Bethesda, MD, USA

**Keywords:** breast cancer, reproductive factors, hormone receptors, histology

## Abstract

Reproductive factors are associated with reduced risk of breast cancer, but less is known about whether there is differential protection against subtypes of breast cancer. Assuming reproductive factors act through hormonal mechanisms they should protect predominantly against cancers expressing oestrogen (ER) and progesterone (PR) receptors. We examined the effect of reproductive factors on subgroups of tumours defined by hormone receptor status as well as histology using data from the NIHCD Women's Contraceptive and Reproductive Experiences (CARE) Study, a multicenter case–control study of breast cancer. We estimated odds ratios (ORs) and 95% confidence intervals (CIs) as measures of relative risk using multivariate unconditional logistic regression methods. Multiparity and early age at first birth were associated with reduced relative risk of ER + PR + tumours (*P* for trend=0.0001 and 0.01, respectively), but not of ER − PR − tumours (*P* for trend=0.27 and 0.85), whereas duration of breastfeeding was associated with lower relative risk of both receptor-positive (*P* for trend=0.0002) and receptor-negative tumours (*P*=0.0004). Our results were consistent across subgroups of women based on age and ethnicity. We found few significant differences by histologic subtype, although the strongest protective effect of multiparity was seen for mixed ductolobular tumours. Our results indicate that parity and age at first birth are associated with reduced risk of receptor-positive tumours only, while lactation is associated with reduced risk of both receptor-positive and -negative tumours. This suggests that parity and lactation act through different mechanisms. This study also suggests that reproductive factors have similar protective effects on breast tumours of lobular and ductal origin.

The epidemiologic evidence that reproductive factors such as number of full-term pregnancies, age at first full-term pregnancy and duration of breastfeeding are strongly predictive of breast cancer risk is substantial (Kelsey *et al*, 1993; Collaborative Group on Hormonal Factors in Breast Cancer, 2002). We recently confirmed these findings in the Women's Contraceptive and Reproductive Experiences (CARE) Study and showed that these effects are similar in African-American and White women (Ursin *et al*, 2004).

Both multiparity and age at first pregnancy may exert their protective effects on breast cancer through hormonal mechanisms (Bernstein *et al*, 1985; Garcia-Closas *et al*, 2002), either through direct action on sex steroids or through hormone-induced breast cell differentiation (Russo *et al*, 1992). The protective effects of lactation may occur because of both hormonal (Petrakis *et al*, 1987; Russo and Russo, 1994) and nonhormonal mechanisms (Murrell, 1991). It has previously been proposed that hormonal breast cancer risk factors ought to be predominantly associated with tumours that express oestrogen (ER) and progesterone (PR) receptors, and less so with receptor-negative tumours (McTiernan *et al*, 1986; Stanford *et al*, 1987; Potter *et al*, 1995; Enger *et al*, 2000; Ursin *et al*, 2002). However, the studies that have addressed the effect of reproductive factors on breast tumours of different receptor status have yielded mixed results (Hildreth *et al*, 1983; McTiernan *et al*, 1986; Stanford *et al*, 1987; Cooper *et al*, 1989; Potter *et al*, 1995; Yoo *et al*, 1997; Huang *et al*, 2000; Britton *et al*, 2002; Cotterchio *et al*, 2003).

The histology of the tumour may complicate this picture even further. It has been hypothesised that lobular cancers may be more strongly associated with certain hormonal risk factors such as postmenopausal hormone therapy use (Li *et al*, 2000; Daling *et al*, 2002). However, this has not been found in all studies (Ursin *et al*, 2002), and there is some evidence that early age at menarche, late age at menopause and obesity are more strongly associated with ductal tumours than lobular tumours (Li *et al*, 2003).

Tumour histology may also be related to receptor status. Lobular tumours are more often receptor positive than ductal tumours (Chu and Anderson, 2002), and it would therefore seem obvious that overall, lobular tumours should be associated with hormonal factors, but once receptor status has been taken into account, it is not clear whether any of the histological subtypes should be more or less associated with hormonal factors.

We combine epidemiological data with data from pathology reports of cases who participated in the Women's CARE Study to examine the relationships of reproductive factors with different types of breast tumours to gain insight into mechanisms that may be operating. If parity and breastfeeding act through hormonal mechanisms, we expect that they will protect predominantly against tumours that express ER and PR (ER + PR + tumours), while if they act through a nonhormonal mechanism, they will be associated with hormone receptor-positive and -negative tumours to an equal extent.

We also evaluate the associations between reproductive factors and development of tumours of different histologies in all women combined as well as by ERPR status.

## MATERIAL AND METHODS

The Women's CARE Study is a population-based case–control study of breast cancer designed to address the role of reproductive, contraceptive and lifestyle factors on the breast cancer risk of White and African-American women ages 35–64 years in five US regions (Atlanta, Seattle, Detroit, Philadelphia and Los Angeles). The design and methods of this study have previously been described in detail (Marchbanks *et al*, 2002).

### Cases

Case patients were diagnosed with pathologically confirmed invasive breast cancer between July 1994 and April 1998, and were identified by the Surveillance, Epidemiology and End Results (SEER) cancer registries in Atlanta, Seattle, Detroit and Los Angeles, or from hospitals in Philadelphia. We restricted eligibility to US-born English-speaking women. We oversampled younger case patients and African-American case patients to maximise their numbers in the study, and we randomly sampled older White case patients to achieve approximately equal numbers of case patients in each 5-year age group. Of the 5982 eligible case patients, we interviewed 4575 (76.5%), including 2953 White patients and 1622 African-Americans.

### Controls

Control subjects were randomly selected from a pool of eligible women identified through random digit dialling (RDD) identified between July 1994 and June 1998. They were US-born English-speaking women who had never been diagnosed with invasive or *in situ* breast cancer. Control subjects were frequency matched to case patients by study centre, race and 5-year age group. We successfully screened 82% of residential households called. Of the 5956 eligible women selected as control subjects, we interviewed 4682 (78.6%), including 3021 White subjects and 1661 African Americans.

### Data collection

Risk factor information was obtained from an in-person interview using a structured questionnaire that included questions on demographics, reproductive history (including breastfeeding), medical history including use of exogenous hormones, family history of cancer and other lifestyle factors. Information was recorded up to the date of diagnosis (month and year) for case patients or the date of initial household contact by RDD for control subjects. All cases and controls were interviewed between September 1994 and December 1998. Informed consent was obtained from all participants. Study protocols were approved by the Institutional Review Board at each participating centre.

### Receptor status

Oestrogen receptor and PR status of case patients was recorded from the pathology record when available at each study centre. All SEER registries routinely collect this information for breast cancer, specifically they collect the laboratory results recorded in the medical records at the time of diagnosis. Overall, 3969 (86.8%) of case patients had ER status data available. Among those missing ER status, the pathology record indicated that the test had not been carried out for 153 cases, it had been ordered but was not available for 171 cases and the information was missing for 252 cases. An additional 30 cases had ER status listed as borderline and were also excluded from these analyses. We had PR information on 3795 women (83.0%). An additional 179 were ordered but results were not available, 238 were not carried out and no information was available on 319. The PR status of 44 was borderline and these were excluded from the analyses. Both ER and PR status were available on 3771 cases, or 82.4%. The percentage of participants with both ER and PR status available by site were: Atlanta 91.5%, Detroit 70.3%, Los Angeles 79.0%, Philadelphia 72.3% and Seattle 93.5%.

### Histology

We conducted separate analyses by histology for 3455 ductal tumours (International Classification of Diseases for Oncology, ICD-O morphology code 8500), 274 lobular tumours (ICD-O morphology code 8520) and 261 mixed ductolobular tumours (ICD-O morphology code 8522). An additional 577 tumours of other histologies were not included in these analyses.

### Analyses

We evaluated the following reproductive variables: gravidity (ever pregnant *vs* never pregnant), parity (no full-term pregnancy *vs* full-term pregnancy, defined as longer than 6 months (>26 weeks)), number of pregnancies, number of full-term pregnancies, age at first full-term pregnancy, years since last full-term pregnancy and duration of breastfeeding.

We excluded 21 women (seven cases and 14 controls) from the analyses because of unknown information on one or more pregnancy or breastfeeding variable These exclusions left us with 4668 controls and 3764 cases for the ERPR analyses. For the histology analyses, we excluded one additional case with missing parity and ERPR information. Thus, we ended up with 3990 cases for the histology analyses. A total of 11 women who had unknown ages at menarche were assigned the median age of 12 years and retained in all analyses.

For each stratum of ERPR status, we estimated odds ratios (ORs) and 95% confidence intervals (CIs) as measures of relative risk using unconditional logistic regression methods (Breslow and Day, 1980), while controlling for a number of potentially confounding variables that we had selected *a prior*i. The following variables were included in all models: age (35–39, 40–44, 45–49, 50–54, 55–59, 60–64 years), race (White and African-American), family history of breast cancer (no first-degree family history, first-degree (mother, sister or daughter) family history, unknown or adopted), age at menarche (⩽11, 12, 13, >13 years), study centre (five sites) and education (⩽high school, technical school or some college, college graduate). Where appropriate, we also adjusted for age at first full-term pregnancy (⩽19, 20–24, 25–29, ⩾30 years) and number of full-term pregnancies (1, 2, 3, 4, ⩾5). For analyses of all women combined, we used never pregnant women as the reference group. For the analyses of age at first birth and total breastfeeding, we only included parous women. The reference group for total breastfeeding was parous women who had never breastfed. All adjustment variables were included as categorical variables in the models. We report the *P*-values from trend tests (Wald statistics) from the case–control analyses in the tables. All *P*-values reported are two-sided.

To determine whether the reproductive factors were associated equally with breast cancer of different receptor statuses, we excluded the controls, and defined ER + PR + cases as ‘cases’ and ER − PR − cases as ‘controls’. We determined the ORs of being a receptor-positive case associated with the various reproductive factors, and estimated the tests for association or trend. A statistically significant test of association or trend (likelihood ratio test) from the case–case analysis suggested that the effect of the exposure factor differed by receptor status. A similar procedure was used for comparing ER + PR − cases and ER − PR + cases with ER − PR − cases, as well as for histology status, where we evaluated whether either lobular or ductolobular cancers differed from ductal tumours.

All analyses were performed using EPILOG (Epicenter Software, Pasadena, CA, USA) or the SAS statistical package (SAS Institute, Cary, NC, USA).

Study protocols were approved by the Institutional Review Boards at each participating centre.

## RESULTS

Cases with data on ER and PR had fewer children (*χ*^2^ *P*=0.01), somewhat later age at first birth (*P*=0.0001), and breastfed longer (*P*=0.03) than those without data on ER and PR (Table 1). Table 2 shows the relative risk estimates of breast cancer associated with reproductive factors by hormone receptor status. In general, results for ER + PR − and ER − PR + tumours showed similar associations to ER + PR + tumours on all of the risk factors shown in the table (*P*-values for all tests comparing ER + PR + , ER + PR − and ER − PR + receptor statuses >0.10, results not shown), we focus our presentation on the differences between the ER + PR + and the ER − PR − cancers. Ever having had a full-term pregnancy was associated with a reduced OR for ER + PR + tumours (OR 0.69, 95% CI 0.59–0.81), while no such effect was observed for ER − PR − tumours (OR 1.05, 95% CI 0.84–1.32, *P* comparing cases by ERPR status=0.0005). Being multiparous was associated with reduced ORs of ER + PR + tumours, but not of ER − PR − tumours (*P* comparing cases by ERPR status=0.00006), with five or more full-term pregnancies conferring an estimated 58% reduction in risk of ER + PR + tumours.

Among parous women, older ages at first birth were associated with modest increases in relative risk estimates of ER + PR + tumours, but not ER − PR − tumours, although the test comparing receptor status did not achieve statistical significance (*P*=0.07).

On the other hand, the effects of breastfeeding did not differ by receptor status. Among parous women, the relative risk estimates for both ER + PR + and ER − PR − tumours were statistically significantly reduced among those who had breastfed compared to those who had not. The effect of total duration of breastfeeding was remarkably similar for both ER + PR + and ER − PR − tumours. Compared to women who never breastfed, women who breastfed 24 months or longer were at an estimated 31 and 35% lower relative risk of ER + PR + and ER − PR − tumours, respectively (*P* comparing cases by ERPR status=0.71).

Table 3 shows the OR per full-term pregnancy as well as the OR per 12 months of breastfeeding by receptor status in different subgroups of women. The results were similar when we restricted the analyses to White women or to African-American women, or when analyses were restricted to specific race–age subgroups. The only exception was in White women under age 50 where multiparity was associated with a statistically significant relative risk reduction for both receptor-positive and -negative tumours (results not shown).

### Histology

In Table 4, we show the effect of reproductive factors on tumours of ductal, ductolobular and lobular histology. Ever having had a full-term pregnancy was associated with an OR of 0.80 (95% CI 0.69–0.92) for ductal tumours, OR of 0.89 (95% CI 0.60–1.33) for lobular tumours and OR of 0.65 (95% CI 0.45–0.93) for ductolobular cancers. Increasing number of full-term pregnancies was associated with a strong protective effect for ductal tumours (*P* for trend=0.00001) and for ductolobular tumours (*P* for trend=0.001). There was no significant protective trend for lobular tumours (*P*=0.19), but having had four or more pregnancies was associated with an OR of 0.56, similar to the OR of 0.62 for ductal tumours and 0.43 for ductolobular tumours. The tests comparing tumours of other histologies with ductal tumours were not statistically significant (all *P*-values=0.20 or higher).

Women with later age at first full-term pregnancy were at higher relative risk of lobular tumours than women with a first full-term pregnancy before age 20 (*P* for trend=0.02); this was not observed for ductal (*P* for trend=0.32) or ductolobular tumours (*P* for trend=0.92). The test for comparing lobular and ductal cancer cases did not reach statistical significance (*P*=0.11).

Total duration of breastfeeding showed slightly stronger risk reductions for ductal than for either ductolobular or lobular cancers, and was statistically significant only for ductal tumours. However, the number of women with long duration of breastfeeding was small in the lobular and ductolobular groups, and the tests comparing lobular or ductolobular to ductal tumours were not statistically significant.

When we did further analyses of tumours of different histologies across different age and ethnic groups, the results were consistent with those reported above (results not shown). When analyses were limited to White women above age 50 with ER + PR + tumours, the only subgroup defined by age, race and receptor status for which there were sufficient number of subjects to analyse histology, the difference between ductolobular and ductal tumours reached marginal statistical significance (OR per FTP for ductal tumours 0.92 (95% CI 0.86–0.99) and OR for ductolobular tumours 0.78 (95% CI 0.65–0.93), *P*=0.05). We observed no statistically significant differences in the relative risks estimates for 12 months of breastfeeding between tumours of the different histologic subtypes (results not shown).

When analyses were restricted to women who had never used hormone therapy, the results were very similar, with no differences between the relative risk estimates of the different histologic types for either full-term pregnancy or breastfeeding.

## DISCUSSION

Our recent evaluation of reproductive factors from the CARE study (Ursin *et al*, 2004) found that the effects of reproductive factors were in general very similar in African-American and White women. In particular, parity and lactation had similar protective effects in the two racial groups.

In the present analyses, we found that multiparity and early age at first birth were associated with reduced relative risk of ER + PR + tumours, but not of ER − PR − tumours. The relative risk reduction observed with duration of breastfeeding did not differ between receptor-negative and -positive tumours. Our results were consistent across subgroups of women based on their age and ethnicity.

Previous studies of the effects of parity or age at first full-term pregnancy (or age at first birth) on tumours of different receptor statuses have been mixed. Two studies found similar effects of parity on receptor-positive and -negative tumours (Yoo *et al*, 1997; Britton *et al*, 2002). One study suggested a protective effect of multiparity on ER + PR + tumours, but not receptor-negative tumours; however, tests comparing these tumour types were not statistically significant, possibly because of few (*n*=80) ER − PR − cases (Potter *et al*, 1995). Another study found similar effects of parity on receptor-positive and -negative tumours among postmenopausal women (Cotterchio *et al*, 2003), while among premenopausal women, the inverse association with parity was only observed for ER + PR + cases. Only one of these previous studies had a sample size greater than ours (Cotterchio *et al*, 2003), but that study had a broader age range (25–74 years), and all results were presented separately for pre- and postmenopausal women. Thus, lack of statistical power in these previous studies may explain why results from our study are only partially consistent with them.

Several studies have addressed the effect of age at first birth on receptor status of breast tumours (Potter *et al*, 1995; Yoo *et al*, 1997; Huang *et al*, 2000; Britton *et al*, 2002; Cotterchio *et al*, 2003). Consistent with our findings, three of these studies found evidence that late age at first birth was associated with receptor-positive tumours only (Potter *et al*, 1995; Huang *et al*, 2000; Cotterchio *et al*, 2003). Our results on breastfeeding are also consistent with the few previous studies that have addressed this (Hildreth *et al*, 1983; McTiernan *et al*, 1986; Cooper *et al*, 1989; Yoo *et al*, 1997; Huang *et al*, 2000; Britton *et al*, 2002; Cotterchio *et al*, 2003).

We had few women with ER − PR + and ER + PR − tumours, but, in general, results for these tumours were similar to those for ER + PR + tumours. Previous reports have not drawn strong conclusions on the associations between these subtypes and reproductive factors (Huang *et al*, 2000; Britton *et al*, 2002). It is possible that some of the similarity between ER + PR + and ER − PR + tumours is due to some ER + PR + tumours that are misclassified as ER − PR + (Horwitz, 1988). By using the combined status of ER and PR, we reduce the problem of including any tumours where one of the receptor statuses was mislabelled.

The finding of a protective effect of pregnancies only on receptor-positive tumours support the evidence suggesting that pregnancies protect against breast cancer primarily through hormonal mechanisms. Pregnancies have been found to reduce plasma levels of oestrogen (oestrone, oestradiol and oestriol) (Bernstein *et al*, 1985; Garcia-Closas *et al*, 2002) and follicular phase levels of progesterone (Garcia-Closas *et al*, 2002), increase levels of sex hormone-binding globulin (Bernstein *et al*, 1985) and modify the oestrogen changes that occur with age (Dorgan *et al*, 1995). Either as a consequence of these changes or of other pregnancy changes, the resulting breast changes are characterised by further differentiation of the terminal duct lobular units (Russo *et al*, 1992).

Lactation has also been proposed to protect against breast cancer through hormonal mechanisms by postponing the resumption of ovulatory menstrual cycles after a pregnancy, by increasing the differentiation of breast tissue (Russo and Russo, 1994) or by altering oestrogen levels in the breast (Petrakis *et al*, 1987). In addition, it has been proposed that lactation also has a direct mechanical effect by which carcinogenic agents are excreted from the breast ductal tissue (Murrell, 1991). Our findings suggest that the effect of lactation may not be hormonal, or that at least the mechanisms for this protection are different from those for pregnancies. For instance, the effect of lactation could still be through hormonal mechanisms if lactation exerts its effect at a time period prior to when the hormonal receptor status of a future tumour is developing.

We found few differences between lobular and ductal tumours. Increasing parity had a greater impact on ductal and ductolobular tumours than on lobular tumours, but age at first full-term pregnancy was slightly more protective against lobular cancers. Thus, overall reproductive factors seem to protect against all three histological types. In general, the findings for ductolobular tumours were more similar to ductal than to lobular tumours. These findings are consistent with a recent gene expression study (Zhao *et al*, 2004), which found that about half of the lobular cancers had similar gene expression patterns to ductal tumours, while the remaining ‘typical’ lobular cancers had a different gene expression pattern. However, in these analyses we found little evidence that these reproductive factors act differently on either ductal or lobular tumours, with the possible exception that early age at first birth was most strongly associated with lower risk of lobular cancers, and that ductolobular tumours were associated with the strongest risk reduction per full-term pregnancy.

Although some evidence exists that lobular tumours are more strongly associated with hormonal risk factors such as postmenopausal hormone use and oral contraceptive use (Li *et al*, 2000; Daling *et al*, 2002) than ductal tumours, this has not been found in all studies (Ursin *et al*, 2002). Further, one study found that early age at menarche, late age at menopause and obesity are more strongly associated with ductal tumours than lobular tumours (Li *et al*, 2003). Our study suggests that ductolobular tumours should either be treated as a separate entity or combined with ductal cancers, but should not be combined with lobular cancers. Further, given the confounding effect of receptor status, studies of histology should also separate tumours by receptor status of tumour.

One limitation of our study is that we used ERPR status as it was reported in the pathology report. Although we presume the majority of cases were assessed by immunohistochemistry, we have no data on the methods used by each laboratory. It is possible that some laboratories used different methods, or that the cutoff for a positive receptor status varied between laboratories. It is also possible that the methods varied geographically, by site. We did adjust all analyses for study site and believe it unlikely that this caused any of the observed associations, and that the most likely effect, if any, would have been to bias our relative risk estimates towards the null value.

Another weakness is that ERPR status was not available for all cases. The frequency of unknown receptor status in our study (17.6%) is remarkably similar to that reported by a prior study conducted within the SEER registries (Chu and Anderson, 2002). In that study of 123 732 tumours, 18% were unknown and 5% were not carried out. The reason for the large number of unknowns is unclear, as is the information on the true distribution of ERPR status among those reported as unknown (Chu and Anderson, 2002). Even though the CARE cases with no ERPR status were more likely to be multiparous, give birth early and have breastfed shorter, we think it is unlikely that receptor information on these cases would have altered the results in our study.

In conclusion, high parity and early age at first birth were associated with a reduction in risk only for ER + PR + tumours. Breastfeeding was associated with a reduction in risk for both ER + PR + and ER − PR − tumours. Combined with previous research, this suggests that parity and age at first birth act through different mechanisms than breastfeeding. All reproductive factors showed similar associations with both ductal, ductolobular and lobular tumours, suggesting that these tumours have similar aetiologies.

## Figures and Tables

**Table 1:** Distribution of reproductive factors among all 4567 breast cancer cases and among the 3764 cases with known tumour oestrogen receptor (ER) and progesterone receptor (PR) status and the 803 cases without known receptor status in the Women's Contraceptive and Reproductive Experiences (CARE) Study

	**All cases**		**Cases with known ERPR status**		**Cases without known ERPR status**		
	***N***	**%**	***N***	**%**	***N***	**%**	***χ*^2^ *P*-value^a^**
Never pregnant	588	12.9	502	13.3	86	10.7	
Pregnant but no full-term pregnancy	299	6.6	249	6.6	50	6.2	
Ever full-term pregnancy	3680	80.6	3013	80.1	667	83.1	0.11
							
*Never pregnant*	588	12.9	502	13.3	86	10.7	
1	770	16.9	633	16.8	137	17.1	
2	1371	30.0	1153	30.6	218	27.2	
3	841	18.4	680	18.1	161	20.1	
4	381	8.3	297	7.9	84	10.5	
5 +	317	6.9	250	6.6	67	8.3	0.01
Pregnant but no full-term pregnancy	299	6.6	249	6.6	50	6.2	
							
**Parous women**							
*Age at first full-term pregnancy*							
⩽19	1044	28.4	806	26.8	238	35.7	
20–24	1367	37.2	1126	37.4	241	36.1	
25–29	777	21.1	667	22.1	110	16.5	0.0001
30 +	492	13.4	414	13.7	78	11.7	
							
*Ever breastfeeding*							
No	1653	44.9	1333	44.2	320	48.0	
Yes	2027	55.1	1680	55.8	347	52.0	0.08
							
*Ever breastfeeding at least 2 weeks*							
Never breastfeeding	1653	44.9	1333	44.2	320	48.0	
Ever breastfeeding <2 weeks	151	4.1	123	4.1	28	4.2	
Ever breastfeeding 2 + weeks	1876	51.0	1557	51.7	319	47.8	0.19
							
*Total breastfeeding (months)*							
0	1653	44.9	1333	44.2	320	48.0	
<1	277	7.5	219	7.3	58	8.7	
1–6	820	22.3	699	23.2	121	18.1	
7–23	663	18.0	550	18.3	113	16.9	
24 +	267	7.3	212	7.0	55	8.3	0.03

a *χ*^2^ *P*-value for the difference between the cases with known and the cases without known ERPR status.

**Table 2:** Odds ratios (OR)^a^ and 95% confidence intervals (CI) of breast cancer of different receptor status associated with reproductive factors

	**ER + PR + (*N*=2130)**				**ER − PR − (*N*=1081)**				**ER + PR − (*N*=368)**				**ER − PR + (*N*=185)**			
**All women**	**Case**	**Control**	**OR**	**95% CI**	**Case**	**Control**	**OR**	**95% CI**	**Case**	**Control**	**OR**	**95% CI**	**Case**	**Control**	**OR**	**95% CI**
*Ever pregnancy*																
No	315	481	1.00		109	481	1.00		52	481	1.00		26	481	1.00	
Yes	1815	4187	0.70	0.60–0.82	972	4187	1.02	0.82–1.28	316	4187	0.71	0.52–0.98	159	4187	0.75	0.48–1.15
*P*-value for comparison to ER − PR − tumours^b^			0.0019								0.085				0.21	
																
*Ever full-term pregnancy*																
Never pregnant	315	481	1.00		109	481	1.00		52	481	1.00		26	481	1.00	
Only non-full-term	158	322	0.77	0.61–0.99	58	322	0.75	0.52–1.06	26	322	0.86	0.52–1.42	7	322	0.36	0.16–0.85
Ever full-term	1657	3865	0.69	0.59–0.81	914	3865	1.05	0.84–1.32	290	3865	0.70	0.51–0.96	152	3865	0.79	0.51–1.23
*P*-value for comparison to ER − PR − tumours			0.0005								0.051				0.27	
																
*Number of full-term pregnancies*																
Never pregnant	315	481	1.00		109	481	1.00		52	481	1.00		26	481	1.00	
1	345	717	0.78	0.64–0.95	183	717	1.07	0.82–1.40	60	717	0.85	0.57–1.27	45	717	1.15	0.69–1.90
2	650	1355	0.74	0.63–0.89	350	1355	1.13	0.89–1.45	102	1355	0.71	0.50–1.02	51	1355	0.72	0.44–1.18
3	380	905	0.65	0.53–0.78	200	905	0.99	0.76–1.30	68	905	0.66	0.45–0.98	32	905	0.72	0.42–1.24
4	159	443	0.55	0.43–0.70	89	443	0.90	0.65–1.24	37	443	0.71	0.45–1.12	12	443	0.56	0.28–1.15
5 +	123	445	0.42	0.33–0.55	92	445	0.94	0.68–1.31	23	445	0.39	0.23–0.67	12	445	0.56	0.27–1.18
Trend *P*^c^			0.0001				0.27				0.0009				0.009	
*P*-value for comparison to ER − PR − tumours			0.00006								0.015				0.04	
																

	**ER + PR + (*N*=1657)**				**ER − PR − (*N*=914)**				**ER + PR − (*N*=290)**				**ER − PR + (*N*=152)**			
**Parous only^d^**	**Case**	**Control**	**OR**	**95% CI**	**Case**	**Control**	**OR**	**95% CI**	**Case**	**Control**	**OR**	**95% CI**	**Case**	**Control**	**OR**	**95% CI**
*Age at first full-term pregnancy*																
⩽19	391	1190	1.00		303	1190	1.00		67	1190	1.00		45	1190	1.00	
20–24	639	1460	1.16	0.99–1.36	314	1460	0.92	0.76–1.10	127	1460	1.32	0.95–1.83	46	1460	0.85	0.54–1.32
25–29	392	718	1.44	1.19–1.75	184	718	1.06	0.84–1.34	58	718	1.23	0.81–1.86	33	718	1.15	0.69–1.94
30 +	235	497	1.22	0.97–1.54	113	497	0.91	0.68–1.22	38	497	1.14	0.70–1.87	28	497	1.17	0.64–2.12
Trend *P*			0.01				0.85				0.59				0.45	
*P*-value for comparison to ER − PR − tumours			0.07								0.66				0.29	
																
*Years since last full-term pregnancy* e																
10 +	1121	2625	1.00		584	2625	1.00		198	2625	1.00		84	2625	1.00	
7–9	85	184	1.21	0.88–1.66	53	184	0.95	0.66–1.38	12	184	1.47	0.72–3.00	5	184	0.55	0.20–1.50
3–6	78	200	1.03	0.73–1.46	54	200	0.86	0.58–1.28	12	200	1.55	0.72–3.34	7	200	0.73	0.28–1.85
<3	25	127	0.55	0.33–0.91	37	127	0.87	0.54–1.40	6	127	1.35	0.49–3.72	9	127	1.42	0.55–3.66
Trend *P*			0.15				0.46				0.32				0.75	
*P*-value for comparison to ER − PR − tumours			0.64								0.17				0.60	
																
*Ever breastfeeding*																
No	717	1533	1.00		422	1533	1.00		130	1533	1.00		64	1533	1.00	
Yes	940	2332	0.78	0.69–0.89	492	2332	0.78	0.66–0.91	160	2332	0.80	0.61–1.03	88	2332	0.92	0.64–1.32
*P*-value for comparison to ER − PR − tumours			0.84								0.61				0.45	
																
*Ever breastfeeding at least 2 weeks*																
Never breastfeeding	717	1533	1.00		422	1533	1.00		130	1533	1.00		64	1533	1.00	
Only <2 weeks	65	150	0.94	0.69–1.29	40	150	0.94	0.65–1.37	9	150	0.74	0.36–1.49	9	150	1.33	0.64–2.76
Ever 2 + weeks	875	2182	0.77	0.68–0.88	452	2182	0.76	0.65–0.90	151	2182	0.80	0.61–1.05	79	2182	0.88	0.61–1.28
*P*-value for comparison to ER − PR − tumours			0.91								0.75				0.58	
																
*Total breastfeeding (month)*																
0	717	1533	1.00		422	1533	1.00		130	1533	1.00		64	1533	1.00	
<1	123	306	0.85	0.67–1.08	62	306	0.76	0.56–1.02	18	306	0.69	0.41–1.15	16	306	1.27	0.72–2.25
1–6	376	902	0.78	0.67–0.91	219	902	0.88	0.73–1.07	66	902	0.80	0.58–1.11	38	902	0.98	0.63–1.50
7–23	323	775	0.79	0.67–0.94	149	775	0.68	0.54–0.85	53	775	0.81	0.57–1.16	25	775	0.74	0.44–1.23
24 +	118	349	0.69	0.54–0.89	62	349	0.65	0.48–0.89	23	349	0.89	0.54–1.47	9	349	0.67	0.31–1.44
Trend *P*			0.0002				0.0004				0.26				0.02	
*P*-value for comparison to ER − PR −			0.71								0.93				0.99	

a ORs are adjusted for categorical variables of age, race, family history of breast cancer, age at menarche, education and study site (see text).

b *P*-value for a test for association or trend (likelihood ratio test) from case–case analyses where ‘controls’ represent ER − PR − cases (see text).

c *P*-value from a test for trend (Wald statistic) from case–control analyses.

d ORs for parous women are also adjusted for number of full-term pregnancies and age at first full-term pregnancy.

e Analyses for years since last full-term pregnancy restricted to women with 2 + full-term pregnancies.

**Table 3:** Adjusted odds ratios (OR)^a^ and 95% confidence intervals (CI) per full-term pregnancy (FTP) and 12 months breastfeeding by oestrogen and progesterone receptor (ERPR) status

	**OR (95% CI)^a^ per FTP**	***P*-value for comparison with ER − PR − cases^b^**	**OR (95% CI)^a^ per 12 months breastfeeding**	***P*-value for comparison with ER − PR − cases^b^**
*All women*				
ER − PR −	0.98 (0.94–1.03)	Comparison group	0.91 (0.84–0.99)	Comparison group
ER + PR +	0.88 (0.85–0.91)	0.00001	0.93 (0.88–1.00)	0.51
ER − PR +	0.89 (0.80–0.99)	0.09	0.82 (0.65–1.04)	0.39
ER + PR −	0.88 (0.82–0.95)	0.009	1.01 (0.89–1.14)	0.43
				
*White women*				
ER − PR −	0.97 (0.91–1.04)	Comparison group	0.87 (0.77–0.97)	Comparison group
ER + PR +	0.87 (0.83–0.91)	0.004	0.92 (0.85–0.99)	0.33
				
*African-American women*				
ER − PR −	0.99 (0.93–1.05)	Comparison group	0.97 (0.85–1.11)	Comparison group
ER + PR +	0.88 (0.83–0.94)	0.002	0.98 (0.87–1.11)	0.89
				
*All women age 50* +				
ER − PR −	1.03 (0.97–1.09)	Comparison group	0.97 (0.85–1.10)	Comparison group
ER + PR +	0.89 (0.85–0.93)	0.0001	0.95 (0.86–1.04)	0.93
				
*All women under age 50*				
ER − PR −	0.93 (0.86–0.99)	Comparison group	0.88 (0.79–0.99)	Comparison group
ER + PR +	0.85 (0.80–0.91)	0.06	0.93 (0.86–1.02)	0.39

a ORs are adjusted for categorical variables of age, race, family history of breast cancer, age at menarche, education and study site (see text). Women with non-full-term pregnancies were not included in these analyses. Only parous women were included in the analyses of breastfeeding.

b *P*-value for a test for trend from a case–case analyses where ‘cases’ represent ER + PR + cases (see text).

**Table 4:** Adjusted odds ratios (ORs)^a^ and 95% confidence intervals (CI) of breast cancer of different histology associated with reproductive factors

	**Ductal (*N*=3455)**				**Lobular (*N*=274)**				**Ductolobular (*N*=261)**			
**All women**	**Case**	**Control**	**OR**	**95% CI**	**Case**	**Control**	**OR**	**95% CI**	**Case**	**Control**	**OR**	**95% CI**
*Ever pregnancy*												
No	441	481	1.00		31	481	1.00		41	481	1.00	
Yes	3014	4187	0.79	0.69–0.91	243	4187	0.91	0.61–1.35	220	4188	0.66	0.46–0.94
*P*-value for comparison to ductal tumours^b^							0.47				0.34	
*P*-value for comparison to ductolobular tumours^c^							0.28					
												
*Ever full-term pregnancy*												
Never pregnant	441	481	1.00		31	481	1.00		41	481	1.00	
Only non-full-term pregnancy	220	322	0.74	0.60–0.92	20	322	1.09	0.60–1.96	20	322	0.75	0.43–1.31
Ever full-term pregnancy	2794	3865	0.80	0.69–0.92	223	3865	0.89	0.60–1.33	200	3866	0.65	0.45–0.93
*P*-value for comparison to ductal tumours							0.55				0.30	
*P*-value for comparison to ductolobular tumours							0.30					
												
*Number of full-term pregnancies*												
Never pregnant	441	481	1.00		31	481	1.00		41	481	1.00	
1	599	717	0.92	0.77–1.09	31	717	0.74	0.44–1.25	43	717	0.76	0.48–1.19
2	1039	1355	0.84	0.72–0.98	86	1355	1.01	0.65–1.55	79	1355	0.70	0.47–1.05
3	631	905	0.75	0.63–0.89	68	905	1.10	0.70–1.73	47	905	0.62	0.40–0.97
4 +	525	888	0.62	0.52–0.74	38	888	0.56	0.33–0.93	31	889	0.43	0.26–0.71
Trend *P*^d^			0.00001				0.19				0.001	
*P*-value for comparison to ductal tumours							0.35				0.24	
*P*-value for comparison to ductolobular tumours							0.20					
												

	**Ductal (*N*=2794)**				**Lobular (*N*=223)**				**Ductolobular (*N*=200)**			
**Parous only^e^**	**Case**	**Control**	**OR**	**95% CI**	**Case**	**Control**	**OR**	**95% CI**	**Case**	**Control**	**OR**	**95% CI**
*Age at first full-term pregnancy*												
⩽19	808	1190	1.00		42	1190	1.00		53	1190	1.00	
20–24	1029	1460	1.01	0.88–1.14	96	1460	1.66	1.11–2.47	71	1461	0.90	0.61–1.33
25–29	576	718	1.13	0.96–1.33	59	718	2.31	1.44–3.68	48	718	1.18	0.75–1.87
30 +	381	497	1.05	0.86–1.27	26	497	1.61	0.89–2.91	28	497	0.90	0.51–1.58
Trend *P*			0.32				0.02				0.92	
*P*-value for comparison to ductal tumours							0.11				0.68	
*P*-value for comparison to ductolobular tumours							0.23					
												
*Ever breastfeeding*												
No	1262	1533	1.00		91	1533	1.00		82	1533	1.00	
Yes	1532	2332	0.80	0.72–0.89	132	2332	0.90	0.67–1.21	118	2333	0.90	0.65–1.23
*P*-value for comparison to ductal tumours							0.41				0.64	
*P*-value for comparison to ductolobular tumours							0.53					
												
*Ever breastfeeding at least 2 weeks*												
Never breastfeeding	1262	1533	1.00		91	1533	1.00		82	1533	1.00	
Ever breastfeeding <2 weeks	115	150	0.94	0.72–1.21	11	150	1.34	0.64–2.41	9	150	1.17	0.57–2.42
Ever breastfeeding 2 + weeks	1417	2182	0.79	0.71–0.88	121	2182	0.87	0.64–1.18	109	2183	0.87	0.63–1.21
*P*-value for comparison to ductal tumours							0.44				0.88	
*P*-value for comparison to ductolobular tumours							0.55					
												
*Total breastfeeding (months)*												
0	1262	1533	1.00		91	1533	1.00		82	1533	1.00	
<1	207	306	0.84	0.69–1.02	21	306	1.34	0.69–1.88	16	306	0.97	0.56–1.71
1–6	608	902	0.80	0.70–0.91	48	902	0.84	0.58–1.22	52	902	0.98	0.67–1.42
7–23	522	775	0.81	0.70–0.94	45	775	0.88	0.59–1.31	31	776	0.68	0.43–1.08
24 +	195	349	0.72	0.59–0.89	18	349	0.83	0.47–1.47	19	349	1.07	0.60–1.91
Trend *P*			0.0001				0.34				0.43	
*P*-value for comparison to ductal tumours							0.56				0.71	
*P*-value for comparison to ductolobular tumours							0.85					

a ORs are adjusted for categorical variables of age, race, family history of breast cancer, age at menarche, education and study site (see text).

b *P*-value for a test for association or trend (likelihood ratio test) from a case–case analyses where ‘cases’ represent cases with ductal tumours (see text).

c *P*-value for a test for association or trend (likelihood ratio test) from a case–case analyses where ‘cases’ represent cases with ductolobular tumours.

d *P*-value from a test for trend (Wald statistic) from case–control analyses.

e ORs for parous women are also adjusted for number of full-term pregnancies and age at first full-term pregnancy.
